# Editorial: Phytochemicals targeting autophagy in treatment of bacterial infection and malignancies

**DOI:** 10.3389/fphar.2023.1205764

**Published:** 2023-05-15

**Authors:** Xiaoxue Bai, Lei Song

**Affiliations:** ^1^ Key Laboratory of Organ Regeneration and Transplantation of the Ministry of Education, Department of General Practice, The First Hospital of Jilin University, Changchun, China; ^2^ Key Laboratory of Organ Regeneration and Transplantation of the Ministry of Education, Center for Pathogen Biology and Infectious Diseases, Department of Respiratory Medicine, The First Hospital of Jilin University, Changchun, China

**Keywords:** cancer, mammalian target of rapamycin (mTOR), AMP-activated protein kinase (AMPK), chemotherapy, apoptosis

Modulation of autophagy in host cells is a crucial strategy for bacterial infections, including those caused by *Legionella*, *Salmonella*, *Streptococcus*, and *Mycobacterium*, among others. It is now known that autophagy plays a dual role in bacterial infections - acting as a double-edged sword (Cheng et al.). On the one hand, autophagy assists in the clearance of intracellular bacteria, but on the other, certain bacteria can hijack the autophagy pathway for their benefit, thus promoting their own survival. Researches indicate that manipulating autophagy could enhance bacterial clearance, which could lead to the development of novel interventions against bacteria that have acquired resistance to antibiotics. For example, the naturally occurring phytochemical capsaicin, found in some chilli plants, may enhance autophagy and improve bacterial clearance in *Shigella flexneri* infected cells (Basak et al.).

In the context of cancer therapy, autophagy plays a divergent role, as it could promote both tumor suppression and tumor progression, depending on the cellular context (Mulcahy Levy and Thorburn, 2020). Autophagy is often necessary for the survival of cancer cells, allowing them to withstand metabolic stress, hypoxia, and chemotherapy-induced death. Thus, autophagy inhibition could serve as a strategy for eradicating cancer cells complementing conventional cancer therapies. Conversely, autophagy activation in cancers like pancreatic cancer can promote tumor suppression. Therefore, understanding the role of autophagy is critical when designing targeted therapies for these diseases.

Phytochemicals, which have bioactive properties, are derived from plants and have previously been shown to prevent or treat a range of ailments, including cardiovascular disease, diabetes, cancer, and neurodegenerative diseases (Wen et al., 2021; Wu et al.). Research over the last decade has also shown that many phytochemicals can modulate autophagy, and thus, there has been growing interest in understanding their potential roles in the treatment of bacterial infections and malignancies. For instance, nordihydroguaiaretic acid, a phenolic lignan from plants belonging to the *Larrea tridentata* family, has been shown to enhance autophagy *in vitro*, increase bacterial clearance in cells infected with *Mycobacterium tuberculosis*, and reduce bacterial colonization of host cells (Guzman-Beltran et al., 2016). This effect is attributed to the increase in the number and size of intracellular autophagic vesicles, which cumulates in the efficient removal of bacterial pathogens from cells (Guzman-Beltran et al., 2016). Curcumin, a polyphenol derived from turmeric, is known to exert anticancer effects via autophagy modulation in addition to myriad other mechanisms, including downregulation of inflammatory markers (Yang et al., 2022). In KRAS mutant colorectal cancer cells, curcumin activates autophagy, induces apoptosis, and enhances the efficacy of chemotherapy agents such as regorafenib by sensitizing cancer cells to death signals (Wu et al.). Similarly, other phytochemicals such as resveratrol have been shown to modulate autophagy in a range of cancer cells and have been evaluated as promising agents for cancer therapy (Jang et al., 2022).

The complex regulation of autophagy involves a range of signaling pathways and proteins, including mammalian target of rapamycin (mTOR), AMP-activated protein kinase (AMPK), and p53, among others (Ryter et al., 2014). In particular, the regulation of autophagy by mTOR is regarded as important, with emerging evidence suggesting that mTOR inhibition could be useful in treating various cancers. Some phytochemicals, such as cinobufagin and berberine, activate autophagy by inhibiting mTOR and activating adenosine monophosphate-activated protein kinase (AMPK) (Huang et al., 2022; Wang et al.). Despite some promising benefits in various laboratory studies, the potential of phytochemicals as modulators of autophagy has not yet been fully explored, and several questions remain unanswered. The possible side effects of phytochemical treatment, for example, are not well documented, and the appropriate dose range for different phytochemicals needs to be established. It is also worth noting that studies of phytochemicals on bacterial infections have generally been limited to *in vitro* studies, making it difficult to extrapolate their findings to *in vivo* situations.

In conclusion, phytochemicals hold enormous promise as autophagy modulators for use in the treatment of bacterial infections and malignancies. Berberine and curcumin are among the phytochemicals that have shown promising results *in vitro* and *in vivo* in laboratory studies of bacterial infections and cancer, respectively. However, many questions remain regarding the potential side effects of phytochemical treatment, appropriate dosing levels, and *in vivo* efficacy. The study of phytochemicals targeting autophagy is still relatively new, and there is much to learn about their therapeutic potential. Further research should involve more extensive human trials and should focus on developing novel interventions that complement conventional treatments for bacterial infections and malignancies. Ultimately, the precise regulation of autophagy in the body could prove clinically indispensable, and phytochemicals will be among the landscape of tools used to achieve this objective. We remain optimistic that phytochemicals targeting autophagy represent an exciting new dimension in medicine that could lead to lasting impact in the fight against bacterial infections and cancer.
